# Defense peptides secreted by helminth pathogens: antimicrobial and/or immunomodulator molecules?

**DOI:** 10.3389/fimmu.2012.00269

**Published:** 2012-08-28

**Authors:** Sophie Cotton, Sheila Donnelly, Mark W. Robinson, John P. Dalton, Karine Thivierge

**Affiliations:** ^1^Institute of Parasitology, McGill University, Sainte-Anne-de-BellevueQC, Canada; ^2^The ithree Institute and The School of Medical and Molecular Biosciences, University of Technology Sydney, UltimoSydney, NSW, Australia; ^3^School of Biological Sciences, Medical Biology Centre, Queen's University BelfastBelfast, UK; ^4^Laboratoire de santé publique du Québec, Institut national de santé publique du Québec, Sainte-Anne-de-BellevueQC, Canada

**Keywords:** defense peptides, helminths, trematodes, parasites, antimicrobial peptides, immunomodulation, innate immune system

## Abstract

Host defense peptides (HDPs) are an evolutionarily conserved component of the innate immune response found in all living species. They possess antimicrobial activities against a broad range of organisms including bacteria, fungi, eukaryotic parasites, and viruses. HDPs also have the ability to enhance immune responses by acting as immunomodulators. We discovered a new family of HDPs derived from pathogenic helminth (worms) that cause enormous disease in animals and humans worldwide. The discovery of these peptides was based on their similar biochemical and functional characteristics to the human defense peptide LL-37. We propose that these new peptides modulate the immune response via molecular mimicry of mammalian HDPs thus providing a mechanism behind the anti-inflammatory properties of helminth infections.

## Introduction

Host defense peptides (HDPs) are conserved in all living species as a primitive component of the innate immune response (Hancock and Diamond, 2000; Bowdish et al., 2004) and have a broad-spectrum of activity against bacteria, fungi, eukaryotic parasites, and viruses (Mookherjee and Hancock, 2007; Andes et al., 2009; Hsu et al., 2009). The abilities of HDPs to suppress infections are mediated by direct antimicrobial properties, modulation of host immune responses, or both (Bommarius et al., 2010). Initially called antimicrobial peptides (AMPs) because of their capacity of directly killing microbes, these peptides are now referred as HDPs due to their added immunomodulatory properties (Hancock and Sahl, 2006).

Helminths comprise a variety of parasitic worms, including nematodes, cestodes and trematodes. We discovered a novel family of HDPs derived from pathogenic trematodes including Fasciola, Schistosoma, Opisthorcis, Paragonimus, and Clonorchis species that cause enormous disease in animals and humans in many parts of the world, particularly in poorer regions. We characterized them as HDPs based on their similar biochemical and functional characteristics to human defense peptides, particularly LL-37 (Robinson et al., 2011). We suggested that these new peptides modulate the immune response via molecular mimicry of HDPs thus providing a mechanism for the anti-inflammatory properties commonly observed in these helminth infections.

## Structural characteristics and properties of HDPs

HDPs are defined as short peptides of 12–50 amino acids with an overall positive charge of +2 to +9 due to the predominance of basic amino acids (arginine, lysine, and histidine) over acidic amino acids (Hancock and Chapple, 1999). They also have the property of folding into amphipathic structures where the charged and hydrophilic portions are segregated from the hydrophobic portions. In general, at least 50% of the amino acids are hydrophobic, allowing interaction with bacterial membranes as a part of HDP mechanism of action (Hancock and Chapple, 1999). In aqueous solution, HDPs remain unstructured but adopt the amphipathic structure upon interaction with membranes (McPhee and Hancock, 2005), an attribute that may be crucial for their activity and for reducing general cytotoxicity (Kindrachuk et al., 2010). Despite their small size and common physico-chemical features, HDPs are classified according to their 3-D structures.

In mammals, the two major families of HDPs include the α- and β-defensins and the cathelicidin. The defensins are characterized by a β-sheet globular structure stabilized by intramolecular disulfide bridges. In human skin, defensins are principally expressed by keratinocytes, neutrophils, and sudoriferous and sebaceous glands (Koczulla and Bals, 2003) where they can be produced constitutively or in response to an inflammatory stimulus. Their expression was reported in other cell types such as tissue macrophages, small intestinal epithelial cells as well as cardiomyocytes. The second group is formed by the cathelicidins which are distinguishable by their linear α-helical structure. All members of the cathelicidin category contain a structurally variable cationic C-terminal portion and a highly conserved N-terminal cathelin domain that must be cleaved to release the active C-terminal peptide (Ramanathan et al., 2002). Cathelicidins are usually expressed by myeloid precursor cells but they are also found in neonatal lymphoid tissue and in mature circulating neutrophils in some species (Zanetti, 2004). In humans, they are produced in epithelial cells and in different tissues and corporal fluids like gastric juices, saliva, semen, sweat, plasma, airway surface liquid, and breast milk (Bals et al., 1998; Murakami et al., 2002; Hase et al., 2003). They are stored in their inactive forms in specific granules and processed exclusively upon stimulation, releasing the active HDPs into the extracellular fluid (Scott et al., 2002; Zanetti, 2004).

The best-characterized cathelicidin is the human LL-37, a cationic (+6) peptide of 37 residues with a molecular mass of 4.5 kDa. It adopts an amphipathic α-helix structure and possesses a broad spectrum antimicrobial activity. This peptide is contained within an inactive secreted precursor protein termed hCAP-18 (human cationic antimicrobial protein 18 kDa; the actual molecular weight is 16 kDa) which is cleaved by endogenous serine proteinase three to release the C-terminal active 37-residue peptide (Agerberth et al., 1995; Gudmundsson et al., 1996). It is produced by neutrophils, macrophages and mucosal epithelial cells upon stimulation by microorganisms and pro-inflammatory mediators (Durr et al., 2006; Mookherjee et al., 2007). Upon injury or infection, there is a strong up-regulation of hCAP-18/LL-37, suggesting the involvement of LL-37 in assisting the immune system. In contrast, several diseases have been associated with the down-regulation of LL-37 such as chronic periodontal disease (Putsep et al., 2002), atopic dermatitis (Ong et al., 2002), chronic ulcers (Heilborn et al., 2003) and an increase of the risk for skin infections. LL-37 also plays a central role in innate immune responses and inflammation. It is known as a potent chemoattractant for mast cells (Niyonsaba et al., 2002), monocytes, T lymphocytes and neutrophils (Yang et al., 2000) through the receptor FPRL1 (formyl peptide receptor-like 1). It promotes wound healing (Heilborn et al., 2003) probably through re-epithelialization and vascularization (Ramos et al., 2011), angiogenesis and arteriogenesis (Ramos et al., 2011) and acts as immune adjuvant (Kurosaka et al., 2005). LL-37 is also known to bind to lipopolysaccharide (LPS) and neutralize its biological actions by preventing its interaction with LPS-binding protein (Larrick et al., 1995; Kirikae et al., 1998; Nagaoka et al., 2001).

## Biological activities of HDPs

The HDPs are multifunctional molecules involved in the direct killing of microbes and in the mediation of various host responses. It is well recognized that HDPs exhibit potent activity against microbes as part of the innate immune system (Auvynet and Rosenstein, 2009). Recent studies also evoke their importance in the regulation of innate immune responses and in protecting against the detrimental effects of an excessive innate inflammatory response (Tecle et al., 2007, 2010; Miles et al., 2009; Murakami et al., 2009; Giuliani et al., 2010).

The antimicrobial activity of HDPs is driven by the charge. The bacterial cell membranes are composed of a high proportion of acidic phospholipids, conferring a negative charge to the surface (Matsuzaki, 1999). The cationic nature of the HDPs is attracted by electrostatic forces to the negative surface of the bacteria, facilitating the direct lysis of the cell through the permeabilization of the membranes (Lehrer et al., 1993). The absence of cholesterol in bacterial membranes also increases the activity of HDPs (Zasloff, 2002). In contrast, the phospholipids in eukaryotic cell membranes are predominantly sequestered in the inner leaflet of the lipid bilayer, leaving the outer leaflet with no or little net charge. Cholesterol is an essential lipid in the composition of eukaryotic membranes, preventing membrane damage. These elements explain why concentrations of HDPs found *in vivo* do not cause host-damage (Boman, 2003). HDPs are usually secreted as cocktails at the site of infection and/or inflammation and act synergistically to increase their effectiveness of antimicrobial activity (Doss et al., 2010; Tecle et al., 2010). While several models on how AMPs actually kill microbes have been proposed (Bierbaum and Sahl, 1985; Westerhoff et al., 1989; Matsuzaki, 1999; Yang et al., 2000; Kragol et al., 2001; Brogden, 2005), direct antimicrobial action is probably not the most important role of HDPs since they present low antimicrobial activities under serum and tissue conditions (Hancock and Diamond, 2000; Hancock, 2001). In fact, it has been reported that some HDPs are inactivated by physiological concentrations of salt and cations when tested *in vitro* and that the physiological concentrations of HDPs are far lower than those required to exert antimicrobial activity *in vitro* (Yang et al., 2002; Boman, 2003; Bowdish et al., 2005).

In addition to their bactericidal activity, accumulating evidences are showing that HDPs also have a key modulatory role in the innate immune response and are an important link between the innate and adaptive immune responses under physiological conditions (Zasloff, 2002). During a microbial invasion, the macrophages and dendritic cells (DCs) of the innate immune system detect the presence of microorganisms through the recognition of specific pathogen-associated molecular patterns (PAMPS) such as the gram-negative LPS endotoxin. An early immune response is driven by the interaction between cell receptors and the PAMPS, leading to the production of potent pro-inflammatory cytokines such as IL-6, IL-12 and TNF (Medzhitov, 2007). The production of these cytokines as well as the up-regulation of co-stimulatory molecules on DCs, macrophages, granulocytes and mast cells, are crucial points in the establishment of a protective adaptive immune response. However, an excessive inflammatory response can lead to sepsis, septic shock and also death (Castellheim et al., 2009; Giuliani et al., 2010). HDPs are known to neutralize LPS-mediated responses (Murakami et al., 2009; Giuliani et al., 2010). They have affinity for LPS and can prevent lethal endotoxemia by suppressing cytokine production by macrophages in the presence of bacteria or other non-specific inflammatory stimuli (Gough et al., 1996; Miles et al., 2009; Tecle et al., 2010). These peptides also participate in the inflammatory response by acting as chemotaxins for immune cells, including the recruitment of neutrophils by an increase of IL-8 production, the mobilization of immunocompetent T-cells and the enhancement of cellular adhesion and the subsequent cellular transepithelial migration (Chertov et al., 1996; Van Wetering et al., 1997; Hata and Gallo, 2008). HDPs promote phagocytosis while inhibiting oxidant responses of neutrophils or monocytes (Tecle et al., 2007; Miles et al., 2009). They also stimulate wound healing and angiogenesis through direct action on epithelial and endothelial cell proliferation (Koczulla and Bals, 2003; Li et al., 2006; Otte et al., 2008). Other activities of HDPs include the modulation of pathways regulating cell survival and apoptosis in various cell types, the induction of chemokines and other immune mediators (Scott et al., 2002; Tjabringa et al., 2003; Bowdish et al., 2004; Mookherjee et al., 2006) and the stimulation of leukocyte degranulation and other microbicidal activities. HDPs have a unique ability to suppress hyperinflammatory responses while maintaining protective effector functions of the immune response.

## Features of helminth-induced immune responses

Although each helminth pathogen triggers characteristic infections associated with the biology of the specific parasite, they all evoke immune responses that share common patterns. The first conserved feature of helminth infection is a T helper (Th) 2-type dominated immune response. Th2-type immunity is typically characterized by an increase in the levels of interleukin-4 (IL-4) and other Th2-type cytokines (including IL-5, IL-9, IL-13, and IL-21), activation and expansion of CD4^+^ Th2 cells, plasma cells secreting immunoglobulin E (IgE), eosinophils, mast cells, and basophils, all of which can produce various types of Th2-type cytokines (Jenkins et al., 2011). The other recurring immunological characteristic of helminth infection is the down regulation of the Th1-type and Th17-type responses and their associated inflammation. Th1-type responses are characterized with increases in the number of Th1 cells, cytotoxic CD8+ T cells, neutrophils and macrophages. Various cytokines that are expressed during Th2-type responses, including IL-4, IL-13, and IL-21 can also downregulate Th1-type and Th17-type.

A further important dimension in helminth infection is the differentiation of alternatively activated macrophages (aaMφs) under the influence of Th2-type cytokines. While aaMφs are recruited to the site of infection and are implicated as functional effectors, they also have strong anti-inflammatory properties and highly express genes whose functions relate to the repair of extracellular matrices, wound healing and fibrosis. The overall outcome of a helminth infection may then be an environment with down-regulated proinflammatory responsiveness, activated damage repair mechanisms and a tightly controlled development of Th2 anti-parasite effector responses (see Cook et al., 2012).

Hence, helminth parasites are master regulators of immune responses utilizing complex mechanisms to favor long-term persistence in the host. Mechanistically, parasite modulation of the immune system is likely to be effected through the release of soluble mediators which ligate, degrade or otherwise interact with host immune cells and molecules (Lightowlers and Rickard, 1988). Much of the earlier literature on immunological effects of helminth products depended on crude extract (such as SEA schistosome eggs antigen), although the degree to which the host is exposed to constituent molecules was uncertain. While both somatically derived and secreted products are known to have immunological activity (Johnston et al., 2009), the secreted helminth modulators are those most likely to be physiological actors at the interface between live parasites and the host (Hewitson et al., 2009). For that reason, research has focused on identifying “excretory-secretory” (ES) products released by live worms with immunomodulatory properties (for reviews on the subject: Maizels and Yazdanbakhsh, 2003; Thomas and Harn, 2004; Dzik, 2006).

## Defense peptides secreted by helminth pathogens

**Figure 1:**
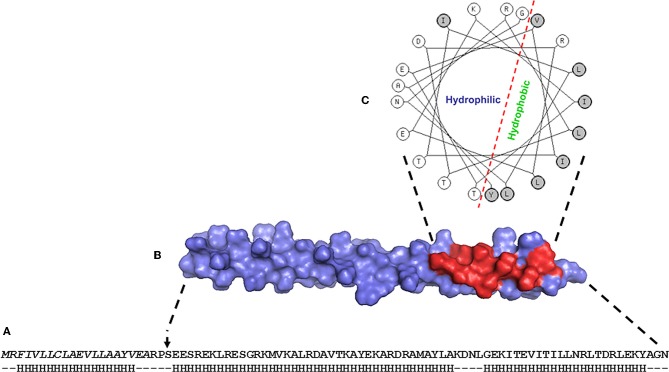
**(A)** Primary amino acid sequence of the archetypal HDM secreted by *Fasciola hepatica*, FhHDM-1. The N-terminal signal peptide is shown in italics and the predicted secondary structure (predominantly alpha helix) is shown below. **(B)** Model structure of FhHDM-1 with the residues forming the hydrophobic face of the molecule shown in red. **(C)** Helical wheel analysis shows that the C-terminal region of FhHDM-1 forms a distinct amphipathic helix.

Prospecting of helminth ES products for molecules with immunomodulatory effects has led us to the discovery of a novel family of proteins, the Helminth Defense Molecules (HDMs). The HDMs were termed following their characterization: they exhibit similar functional and biochemical properties to the human defense peptides, defensins and cathelicidins. To date, the best studied HDM is the 8 kDa protein (FhHDM-1) secreted by the trematode, *Fasciola hepatica*, which causes liver fluke diseases in animals and humans. FhHDM-1 can be grouped in the cathelicidin family as it has a high propensity to adopt α-helical secondary structure (Figure 1). In addition, similar to hCAP18, the secreted FhHDM-1 undergoes cleavage by an endogenous protease (the major cysteine protease from *F. hepatica*, cathepsin L1) to release a C-terminal fragment. The 34-residue C-terminal peptide of FhHDM-1 contains a 21-residue amphipathic helix which exhibits a marked structural parallel with the bioactive human LL-37 peptide. The amphipathic helix of LL-37 anchors the peptide to phospholipid membranes through interaction with hydrophobic face (Agerberth et al., 1995; Porcelli et al., 2008) and is important for its antimicrobial activity (Giuliani et al., 2010). The same amphipathic helix of the cathelicidin hCAP18-derived peptide has also been suggested to be responsible for interacting with LPS (Hoess et al., 1993; Porro, 1994). Like LL-37, the amphipathic helix of the C-terminal peptide of FhHDM-1 binds *E. coli* LPS; it is a key functional determinant necessary for its biological properties (Robinson et al., 2011). Phylogenetic/bioinformatic studies revealed that a family of related HDMs are expressed by several major animal and human trematodes that inhabit various tissues of the host including the mesenteric blood vessels (Schistosoma), the liver (Fasciola, Opisthorcis, Clonorchis) and lungs (Paragonimus).

## Why would parasites have a need for defense peptide?

It is well known that intestinal injury and systemic endotoxemia are two factors leading to morbidity in helminth infected mice (Herbert et al., 2004; Leeto et al., 2006). Loss of gut barrier function and consequently the migration of luminal antigens (bacteria and their toxic products) into the systemic circulation are frequent in helminth infection. Accordingly, enteric nematode infection is characterized by enhanced permeability of the intestinal epithelium, primarily mediated by activated mast cells (McDermott et al., 2003), which contributes to parasite rejection but may lead to the translocation of bacterial LPS into the portal circulation (Farid et al., 2008). The same phenomena can be observed in non-enteric worms. For instance, both *Schistosoma mansoni* (a trematode that resides in the mesenteric vein) and *F. hepatica* (a trematode that lives in the bile ducts) cause damages leading to the systemic movement of bacteria (Ogunrinade and Adegoke, 1982; Herbert et al., 2004; Ferraz et al., 2005; Valero et al., 2006). Despite this translocation of enteric microbes, fatal endotoxemia during infection with trematodes is not a frequent situation (Onguru et al., 2011). Additionally, in endemic areas for helminth parasites, co-infection with gram-negative bacteria, most commonly *Salmonella* sp, is frequent (Melhem and Loverde, 1984). The mechanisms of resistance to septicaemia during helminth infection are not fully understood. One explanation proposed by Robinson et al. (2011) is that the active secretion of HDM by parasites during their lifespan in the mammalian host ensures that potentially lethal LPS, either from intestinal flora or from microbial co-infections, is neutralised and that LPS-mediated activation of macrophages is controlled. In fact, bacterial LPS is known to be a key molecule in the pathogenesis of endotoxin shock associated with gram-negative bacterial infections (Lehmann et al., 1987; Morrison et al., 1994; Castellheim et al., 2009). As mentioned in Section “Structural characteristics and properties of HDPs,” LL-37 neutralises the biological activities of LPS by binding to the microbial molecule (Kirikae et al., 1998). The transfer of LPS to cellular CD14 by serum LPS-binding protein (LBP) is the first event in the recognition of microbial infection. This bimolecular complex then initiates downstream signaling via interaction with cellular TLRs (Beutler et al., 2003), which results in the secretion of inflammatory mediators. Just like human LL-37, the direct binding of FhHDM-1 to LPS blocks the interaction of LPS with LBP, thus reducing the number of LPS molecules that are targeted to the TLR signaling complex on the macrophage cell surface. This in turn prevents LPS-induced activation of macrophages. Therefore, FhHDM-1 impairs LPS signaling and protect against harmful immune responses by reducing the release of inflammatory mediators from macrophages.

## Conclusion

HDPs are an evolutionarily conserved component of the innate immune response and are found among all classes of life. They have been demonstrated to possess antimicrobial activities on a broad range of organism, killing Gram negative and Gram positive bacteria, mycobacteria, enveloped viruses, fungi and even transformed or cancerous cells. It also appears that HDPs have the ability to enhance immunity by functioning as immunomodulators. The discovery of a family of defense peptides that is conserved amongst medically-important trematode pathogens has raised the question of their utility for helminths. Why would parasitic worms need HDPs? Helminth parasites are master regulators of immune responses in order to ensure life-long persistence in the host. One strategy of immune regulation is the secretion of a wide range of immunoregulatory molecules, which are able to target various host cells and alter them to induce a highly directed host response known as a “modified Th2-type response.” Our recent finding of a family of HDMs that modulate the immune response via molecular mimicry of HDPs provides a common mechanism for the anti-inflammatory properties of helminth infection (Robinson et al., 2011). By targeting key stages in LPS-mediated cell signaling, the helminth parasite prevents the activation of innate immune response and enhances its longevity by increasing the survival of the host.

### Conflict of interest statement

The authors declare that the research was conducted in the absence of any commercial or financial relationships that could be construed as a potential conflict of interest.
